# Patients’ self-management of adverse events and patient-reported outcomes in advanced renal cell carcinoma treated with targeted therapies: A prospective, longitudinal, observational study

**DOI:** 10.1186/s41687-022-00532-0

**Published:** 2022-12-16

**Authors:** Sung-Hoo Hong, Ho Seok Chung, Ill-Young Seo, Tae Gyun Kwon, Hyeon Jeong, Jae-Il Chung, Seung Hyun Jeon, Jae Young Park, Hong Koo Ha, Byung-Ha Chung, Wan Song, Young-Joo Kim, Sang-Hee Kim, Jee-Sun Lee, Juneyoung Lee, Jinsoo Chung

**Affiliations:** 1 Department of Urology, The Catholic University of Korea Seoul St. Mary’s Hospital at Seocho-gu, Seoul, Republic of Korea; 2 Department of Urology, Chonnam National University Hwasun Hospital at Hwasun-gun, Jeollanam-do, Republic of Korea; 3 Department of Urology, Wonkwang University Hospital at Iksan, Jeonlabuk-do, Republic of Korea; 4 Department of Urology, School of Medicine, Kyungpook National University, Daegu, Republic of Korea; 5 Department of Urology, SMG-SNU Boramae Medical Center at Dongjak-gu, Seoul, Republic of Korea; 6 Department of Urology, Inje University Busan Paik Hospital, Busanjin-gu, Busan, Republic of Korea; 7 Department of Urology, Kyung Hee University School of Medicine at Dongdaemun-gu, Seoul, Republic of Korea; 8 Department of Urology, Korea University Ansan Hospital at Ansan-si, Danwon-gu, Gyeonggi-do, Republic of Korea; 9 Department of Urology, Pusan National University Hospital at Seo-gu, Busan, Republic of Korea; 10 Department of Urology, Gangnam Severance Hospital, Yonsei University Health System at Gangnam-gu, Seoul, Republic of Korea; 11 Department of Urology, Samsung Medical Center, Sungkyunkwan University School of Medicine, Seoul, Republic of Korea; 12 Medical Division, Pfizer Biopharmaceuticals Group, Pfizer Pharmaceuticals Korea Limited, Seoul, Republic of Korea; 13 Department of Biostatistics, College of Medicine, Korea University, Seoul, Republic of Korea; 14 Center for Urologic Cancer, National Cancer Center, Goyang, Republic of Korea

**Keywords:** Self-management, Side effects and adverse reactions, Molecular targeted therapy, Carcinoma, Renal cell, Patient reported outcome measures

## Abstract

**Background:**

Early intervention to reduce the impact of adverse events (AEs) may improve patients’ quality of life and enable optimal treatment duration.

**Methods:**

This nationwide, multicenter, prospective, longitudinal, 1-year observational study investigated patients’ self-management of AEs associated with targeted therapy for advanced renal cell carcinoma (RCC) and explored corresponding outcomes, including treatment duration and patient-reported outcomes (PROs).

**Results:**

We enrolled 77 advanced RCC patients (mean age 62 years) treated with a first targeted therapy. 210 cases of seven AEs of interest (fatigue, hand-foot syndrome, oral mucosal inflammation, diarrhea, gastrointestinal symptoms, hypertension, and anorexia) were observed. Most AEs were mild to moderate. Overall, 63.4% of patients were identified as managing their AEs well, reporting numerically longer treatment duration and significantly higher PRO scores than patients identified as poor managers.

**Conclusions:**

Longer treatment duration and improved PROs were observed when advanced RCC patients managed targeted therapy-associated AEs well. Repeated education for consolidating AE self-management could be considered to enhance overall treatment outcomes.

## Background

Continuous evolution in the treatment of advanced renal cell carcinoma (RCC) has helped to reduce the disease burden. First-line systemic therapies have broadened in terms of numbers and mechanisms of actions. The most recent change in advanced RCC treatment was the introduction of immune checkpoint inhibitors, which alter the interaction between immune cells and antigen-presenting cells [1]. Over the past decade, since before the era of immune checkpoint inhibitors until the current treatment era, targeted therapy has been the standard of care for the treatment of metastatic RCC (mRCC). Since 2005, the advent of targeted therapy has led to a revolution in mRCC treatment. Consequently, the treatment paradigm has shifted dramatically in real-world clinical practice. Single-agent tyrosine kinase inhibitors (TKIs), vascular endothelial growth factor inhibitors (including pazopanib, sunitinib, axitinib, and cabozantinib), and temsirolimus, which targets the mammalian target of rapamycin pathway, are still recommended as alternative first-line and/or subsequent therapies for mRCC [1].

Prolonging survival and simultaneously maintaining good health-related quality of life (HRQoL) are the ultimate objectives of mRCC treatment. As targeted therapy has achieved survival prolongation in patients with mRCC, HRQoL has received increasing attention from healthcare providers to achieve the optimal treatment goal. Along with the severe symptoms of cancer itself, adverse events (AEs) related to cancer therapies may affect the HRQoL of patients [2]. Unlike AEs related to cytotoxic agents, AEs associated with targeted agents are mostly mild to moderate and are well managed with supportive measures [3]. Early intervention to reduce those AEs may improve HRQoL and enable an acceptable duration of therapy [3]. In real clinical practice, healthcare practitioners provide education to patients with respect to self-management of AEs related to targeted agents, as part of early intervention. A previous study on the recommendations for AE management from patients’ perspectives, reported positive experiences of patients regarding recommendations for AE self-management [4].

As few studies have been conducted on the self-management of AEs in advanced RCC, this study primarily aimed to investigate AEs including grades and status of self-management of AEs related to targeted therapy in patients with advanced RCC in Korea. Additionally, this study compared treatment duration and patient-reported outcomes (PROs), including symptom burden and HRQoL, according to the status of AE self-management.

## Materials and methods

### Study design

This nationwide, multicenter, prospective, longitudinal, 1-year observational study, was conducted from October 2016 to October 2019 in urology departments at 12 representative hospitals which treat advanced RCC in Korea.

### Participants and sample size

Patients who met the following criteria were eligible: (i) diagnosis of advanced RCC (metastatic RCC including lymph node metastases which are eligible for targeted systemic treatment), (ii) age ≥ 19 years, (iii) treatment with first targeted therapy, including initiation of targeted therapy after cytokine therapy and other cancer therapy, (iv) newly experienced one of seven AEs of interest which need self-management by patients (fatigue, hand-foot syndrome, oral mucosal inflammation, diarrhea, gastrointestinal symptoms, hypertension, and anorexia), and (v) received education for self-management of AEs from a medical team. Patients who were hospitalized or treated with immunotherapy or other systemic chemotherapy were excluded. Only patients who had read and signed the written consent forms before study participation were enrolled.

As the aim of this study was a description of AEs including grades and patients’ self-management of AEs, the sample size was calculated using a precision analysis. Based on the published result [5], we assumed 4.9 as a standard deviation (sd) of number of AEs. With ± 1.1 error margin (d), a total of 77 subjects (***n***′) are needed to maintain 95% confidence level, based on an appropriate formula of **(*z***_***1-a***_^**2**^** x *sd***^**2**^**)/*d***^**2**^ [6]. For the estimated sd of AE, the highest value amongst reported information on angiogenesis inhibitors (for sunitinib) was used to determine our study size conservatively, which was for all targeted therapies. Further, the total number of patients in Korea can be projected as 4,000 and, hence, the targeted number of study subjects was adjusted as 75 patients by multiplying an adjustment factor (**1 − *n***′**/4000**). Considering a 5% follow-up loss rate, this study aimed to enroll a total of 79 subjects who satisfy eligibility criteria.

### Data collection and measurements

The institutional review board at each participating center approved this study.

Patients were observed for 1 year from enrolment.

Data on patients’ demographics, clinical characteristics, and treatments were collected through medical chart review. Some of the time-dependent variables, including response criteria, Eastern Cooperative Oncology Group Performance Status (ECOG PS), metastasis site, treatment schedule change (changes of dose and/or of follow-up schedule), and admission, were collected at baseline, 6 months, and 12 months of follow up.

Data on AEs, AE grades, and patients’ self-management were collected through a patient survey. Data regarding AE events and patients’ self-reported grades were collected at baseline and at the subsequent hospital follow-up visits after the AE occurrence during the 1-year observation. Questionnaires for AE cases and self-reported AE grades were developed based on Common Terminology Criteria for Adverse Events [7]. Patients firstly tick `yes/no’ to questions on the occurrence of the seven AEs of interest then, if they answered `yes’, patients tick the statement which is best explains the grade of the AE. For example, if the patient ticked `yes’ for anorexia, then the patient would tick one of the following: (1) Loss of appetite without alteration in eating habits, (2) Oral intake altered without significant weight loss or malnutrition, (3) Associated with significant weight loss or malnutrition, (4) Life-threatening consequences. Due to variation in the scheduling of follow-up visits within real-word clinical practice, the time point for follow-up data collection at 6 and 12 months from baseline had a + / − 8-week window period to cover more patients' visits. PRO and HRQoL measures applied to all participating patients when they visited the hospitals. Data on patients’ self-management were collected through a patient survey questionnaire developed by the study team (Table 1). Patients answered each question with four options: very poor, poor, well, and very well.

**Table 1:** Questionnaire on patients’ self-management for the adverse events Guide: Please answer the Questions relating to the adverse events that you reported at your previous hospital visit^a^

Adverse event	Questions on patients’ self-management	Options to answer
Fatigue	I have tried to lead a well-regulated life to control my fatigue	Very poor Poor Well Very well
Hand-foot syndrome	I have tried to moisturize my hands and feet by applying lotion and taken the prescribed medicines compliantly	
Oral mucosal inflammation	I have managed my oral condition by gargling and taken the prescribed medicines compliantly	
Diarrhea	I have paid attention to my eating habits and taken medicines if needed	
Gastrointestinal symptoms	I have drunk sufficient water, fulfilled a well-regulated eating habit, and taken the prescribed medicine compliantly	
Hypertension	I have regularly checked my blood pressure and taken the prescribed medicine compliantly	
Anorexia	I have eaten soft food or had frequent small meals and taken nutritional supplements as recommended	

^a^The patient survey of AEs was produced in paper format. The form was horizontally long and folded into sections divided for each visit. Patients were able to write the content of their current (next) visit whilst referring to their previous responses on the survey form

Symptom burden and HRQoL were surveyed using validated instruments and measured at baseline, next follow up, 6 months, and 12 months visits. The EuroQol-Five Dimensional-Five Level (EQ-5D-5L), a generic HRQoL instrument that has been used widely for utility-based HRQoL measurements, was applied. EQ-5D-5L comprises five domains (mobility, self-care, anxiety/depression, usual activities, and pain/discomfort), with five responses (no, slight, moderate, severe, and extreme problems) [8]. The validated Korean version of the EQ-5D-5L was used and we employed the Korean value set for score conversion to the index value [9]. The EuroQoL-visual analog scale (EQVAS) measures self-rated health on a vertical scale, with scores ranging from 0 (worst imaginable health status) to 100 (best imaginable health status). High scores on both EQ-5D-5L and EQVAS correspond to enhanced HRQoL.

The Korean version of the Functional Kidney Symptom Index-15 (FKSI-15) was utilized to measure the symptom burden of patients with advanced RCC. The Korean version of the FKSI-15 was translated from the English version following established Functional Assessment of Chronic Illness Therapy (FACIT) multilingual translation methodology, which was developed and validated to ensure that resulting translations of quantitative measures reflect conceptual equivalence with the source document rendered in language that is culturally acceptable and relevant to the target population [10–12]. FKSI-15 comprises 15 questions regarding reliable and valid symptom indices for evaluating kidney cancer patients (e.g. I have a lack of energy, I have pain, and I am losing weight, etc.), with five response scales (never, slightly, moderately, fairly, and extremely experienced). The total FKSI-15 score ranged from 0 to 60, in which 0 indicates severely symptomatic and 60 corresponding to asymptomatic [13].

### Statistical analysis

**Fig. 1:**
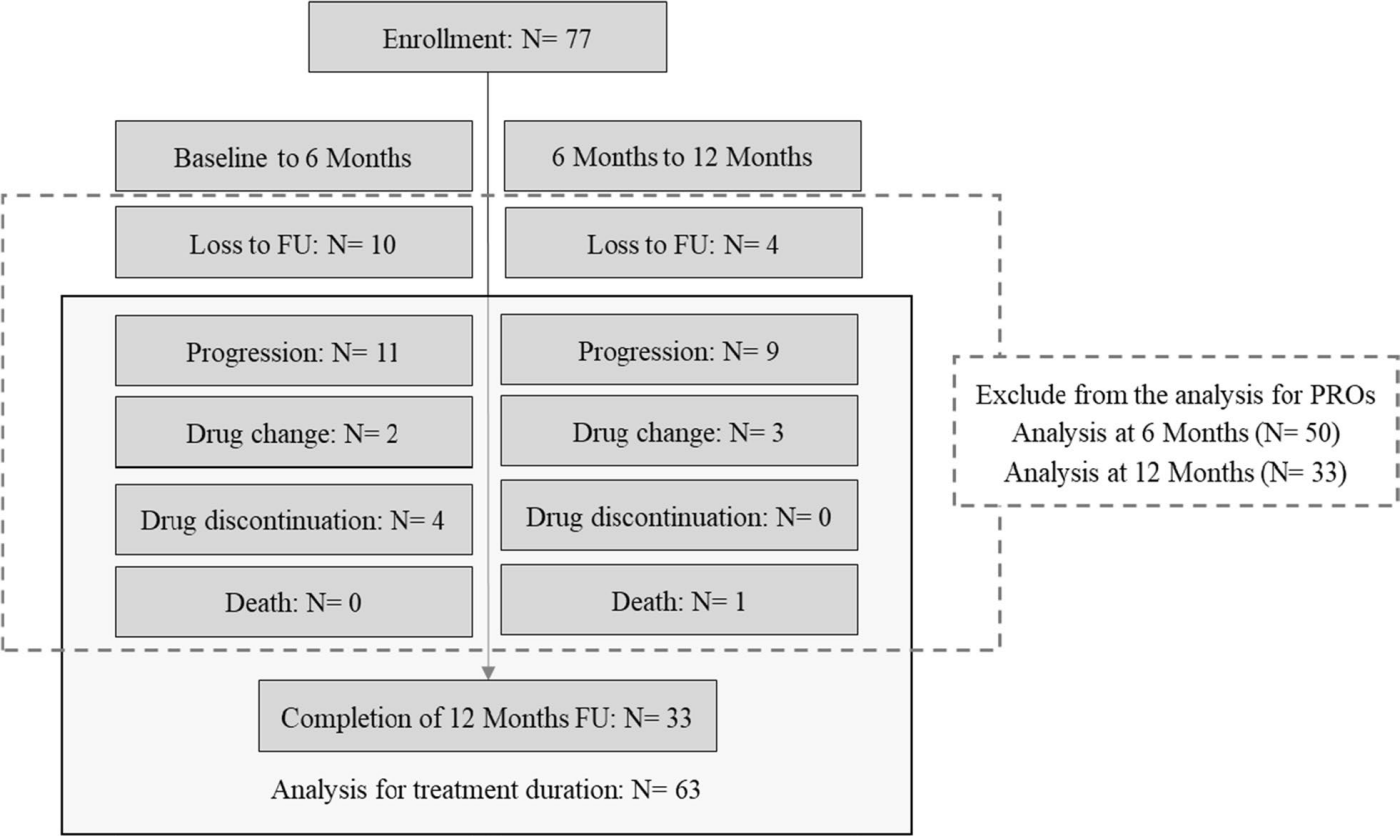
Flow chart of patient numbers included in each analysis

Patients included in each analysis are shown in Fig. 1. During the observational period, patients who died (N = 1), had disease progression (N = 20), were lost to follow-up (when patients visited the hospital outside the follow-up time points, including window periods) (N = 14), changed treatment to immunotherapy (N = 5), drug discontinuation (N = 4), and newly participated in another interventional study (N = 0), were excluded from the analysis for PROs in this study.

Demographic and clinical characteristics of the study participants, and characteristics of AEs, are presented as frequency (percentage) for categorical variables and mean (sd) for numeric variables. Differences of the repeated-measure outcomes EQ-5D-5L, EQVAS, and FKSI-15 at each time point, were statistically tested with a linear mixed model.

Self-management was defined as “well managed” if the patient responded either “well” or “very well” in the questionnaire for all AEs experienced during the study period, and as “poor managed” if at least one experienced AE had a response of “poor” or “very poor.” Treatment duration was calculated from the date of targeted therapy initiation to the date of last follow-up visit. In univariate analysis, treatment duration and PROs were compared according to the patients’ self-management status of AEs using Student’s t-test or the Mann–Whitney U-test.

Confounders associated with treatment duration and PROs were chosen based on p < 0.1 in univariate analysis. Other than the selected confounding variables from the univariate analysis, age, sex, Charlson comorbidity index, and AEs, were fixed as confounding variables in all models. In multivariable analysis, linear regression was performed for treatment duration. For FKSI-15, EQ-5D-5L, and EQVAS, sequential conditional mean models (SCMMs) were employed. Namely, variables of preceding and current measurements of confounders as time-varying covariates, as well as preceding PRO measurements, were adjusted, following the fourth model of the SCMMs described by Keogh et al. [14].

All statistical analyses were performed using SAS software version 9.4 (SAS Institute, Cary, NC, USA), and a two-sided p < 0.05 was considered the minimum level of statistical significance.

## Results

### Demographic and clinical characteristics

Patient demographics and clinical characteristics are shown in Table 2. A total of 77 patients with advanced RCC were enrolled. At baseline, the mean patient age was 62 years, 72.7% patients were male, and 40.3% patients had comorbidities. Overall, most (96.1%) patients had clear-cell RCC and 64.9%, 20.8%, and 18.2% had metastasis to the lung, bone, and lymph nodes, respectively. All patients had grade 0 or 1 ECOG PS. The mean duration of advanced RCC was approximately 30 months and patients were treated with targeted therapy for a mean of about 12 months. Most (82.4%) patients had stable disease, 8.1% had a partial response, and 9.5% had a complete response at baseline of the targeted therapy (Table 2). 50 and 33 patients were followed up at 6 months and 12 months, respectively. Among patients who remained under study observation, a treatment schedule change was noted in 11 patients (Table 2).

**Table 2:** Demographics and clinical characteristics of patients

	Baseline (N = 77)	6 months (N = 50)	12 months (N = 33)
Sex, N (%)			
Male	56 (72.7)		
Female	21 (27.3)		
Age (years), mean (SD)	62.29 (10.3)		
BMI (kg/m^2^), mean (SD)	24.47 (3.7)		
Disease duration (days), mean (SD)	899.6 (1087.5)		
Treatment duration (days), mean (SD)	343.60 (526.4)		
Histology, N (%)			
Clear	74 (96.1)		
Non-clear	3 (3.9)		
Comorbidity, N (%)^a^			
Yes	31 (40.3)		
No	46 (59.7)		
CCI score, mean (SD)	0.2 (0.4)		
Surgery, N (%)	31 (40.3)		
Radiation therapy, N (%)	6 (7.8)		
Immunotherapy, N (%)	2 (2.6)		
Other chemotherapy, N (%)	1 (1.3)		
Response criteria, N (%)			
Stable disease	61 (82.4)	30 (60.0)	22 (66.7)
Partial response	6 (8.1)	17 (34.0)	9 (27.3)
Complete response	7 (9.5)	3 (6.0)	2 (6.1)
ECOG PS, N (%)			
0	58 (75.3)	39 (78.0)	26 (78.8)
1	19 (24.7)	11 (22.0)	6 (18.2)
2–4	0 (0.0)	0 (0.0)	1 (3.0)
Metastasis site, N (%)			
Lung	50 (64.9)	31 (62.0)	20 (60.6)
Bone	16 (20.8)	12 (24.0)	7 (21.2)
Liver	1 (1.3)	1 (2.0)	1 (3.0)
Lymph node	14 (18.2)	9 (18.0)	6 (18.2)
Soft tissue	0 (0.0)	1 (2.0)	0 (0.0)
Brain	2 (2.6)	1 (2.0)	0 (0.0)
Spine	2 (2.6)	2 (4.0)	2 (6.1)
Skin	0 (0.0)	0 (0.0)	0 (0.0)
Pancreas	4 (5.2)	3 (6.0)	3 (9.1)
Others	9 (11.7)	6 (12.0)	5 (15.2)
Treatment schedule change, N (%)		5 (10.0)	6 (18.2)
Admission, N (%)^a^	–	5 (10.0)	2 (6.1)

BMI, body mass index; CCI, Charlson comorbidity index; ECOG PS, Eastern Cooperative Oncology Group performance status; N, number; SD, standard deviation. ^a^Total admission, N = 12

### AEs and patients’ self-management of AEs

Overall, 210 cases of the seven AEs of interest were observed, and a mean of 2.8 (sd 1.6) cases of AEs were experienced per person. Of the total cases, 201 were observed at baseline and additional 9 cases were captured during follow up. Fatigue (23.8%) was the most frequent AE followed by anorexia (15.7%) and diarrhea (14.3%). With respect to severity, most AEs (91%) were mild to moderate, whereas 20 cases were severe (Table 3). Beyond the seven AEs of interest, 21 cases of other AEs were observed, consisting of mostly mild to moderate skin, oral, and urination symptoms; hair color change; pain; oedema; and dizziness (data not shown in tables).

**Table 3:** Adverse events and patients’ self-management

	Total	Fatigue	Hand-foot syndrome	Oral mucosal inflammation	Diarrhea	Gastrointestinal symptoms	Hypertension	Anorexia
Frequency of AEs, N (%)	210 (100.0)	50 (23.8)	25 (11.9)	25 (11.9)	30 (14.3)	23 (11.00)	24 (11.4)	33 (15.7)
95% C.I.*		(18.22, 30.16)	(7.85, 17.07)	(7.85, 17.07)	(9.85, 19.76)	(7.07, 15.98)	(7.46, 16.53)	(11.07, 21.35)
AE grades, N (%)								
1 Mild	122 (58.1)	34 (68.0)	8 (32.0)	12 (48.0)	24 (80.0)	18 (78.3)	14 (58.3)	12 (36.4)
2 Moderate	68 (32.4)	12 (24.0)	14 (56.0)	13 (52.0)	4 (13.3)	3 (13.0)	8 (33.3)	14 (42.4)
3 Severe	20 (9.5)	4 (8.0)	3 (12.0)	0 (0.0)	2 (6.7)	2 (8.7)	2 (8.3)	7 (21.2)
4, 5 Life-threatening & Death-related	0 (0.0)	0 (0.0)	0 (0.0)	0 (0.0)	0 (0.0)	0 (0.0)	0 (0.0)	0 (0.0)
AE management, N (%)								
Very poor	5 (2.6)	2 (4.4)	1 (4.4)	0 (0.0)	2 (6.9)	0 (0.0)	0 (0.0)	0 (0.0)
Poor	37 (19.2)	10 (21.7)	3 (13.0)	5 (21.7)	3 (10.3)	4 (19.1)	5 (23.8)	7 (23.8)
Well	141 (73.1)	33 (71.7)	19 (82.6)	17 (73.9)	23 (79.3)	15 (71.4)	12 (57.1)	22 (57.1)
Very well	10 (5.2)	1 (2.2)	0 (0.0)	1 (4.4)	1 (3.5)	2 (9.5)	4 (19.1)	1 (19.1)
AE grade changes, N (%)								
Worsened	13 (6.7)	5 (10.9)	2 (8.7)	1 (4.3)	0 (0.0)	1 (3.6)	1 (3.7)	3 (8.6)
Steady	114 (58.8)	24 (52.2)	14 (60.9)	15 (65.2)	19 (65.5)	12 (42.9)	15 (55.6)	15 (42.9
Improved	27 (13.9)	6 (13.0)	4 (17.4)	5 (21.7)	3 (10.3)	8 (28.6)	6 (22.2)	12 (34.3)
Resolved	40 (20.6)	11 (23.9)	3 (13.0)	2 (8.7)	7 (24.1)	7 (25.0)	5 (18.5)	5 (14.3)
Grades of remaining AEs^†^, N (%)								
1 Mild	96 (62.3)	21 (60.0)	8 (40.0)	15 (71.4)	20 (90.9)	12 (85.7)	10 (58.8)	10 (40.0)
2 Moderate	53 (34.4)	14 (40.0)	8 (40.0)	6 (28.6)	2 (9.1)	2 (14.3)	7 (41.2)	14 (56.0)
3 Severe	5 (3.3)	0 (0.0)	4 (20.0)	0 (0.0)	0 (0.0)	0 (0.0)	0 (0.0)	1 (4.0)
4, 5 Life-threatening & Death-related	0 (0.0)	0 (0.0)	0 (0.0)	0 (0.0)	0 (0.0)	0 (0.0)	0 (0.0)	0 (0.0)

AE, adverse event; N, number of cases. * C.I.: Clopper-Pearson exact confidence interval. ^†^AEs for which symptoms remain

Of the seven AEs of interest, 73.1% and 5.2% of the AE cases were managed `well’ and `very well’, whereas 19.2% and 2.6% of cases were managed `poorly’ and `very poorly’, respectively. At the next follow-up (median 28 days; min 12 ~ max 84 days), 40 cases (20.6%) were resolved and 27 cases (13.9%) had improved symptom grade. Among all AE cases, gastrointestinal symptoms (53.6%) and anorexia (48.6%) showed the greatest proportions of improvement or remission. Conversely, 13 (6.7%) cases worsened in symptom grade, of which fatigue was the most common (10.9%); 58.8% of all AEs remained the same (Table 3).

With respect to our definition of patients’ management of AEs, 63.4% of patients were classified into the `well managed’ group. Treatment schedule changes were numerically more frequent in the `poor managed’ group compared to the well managed group (25% vs 17%, *p* = 0.505) (data not shown in tables).

### Treatment duration and PROs according to patients’ self-management of AEs

**Fig. 2:**
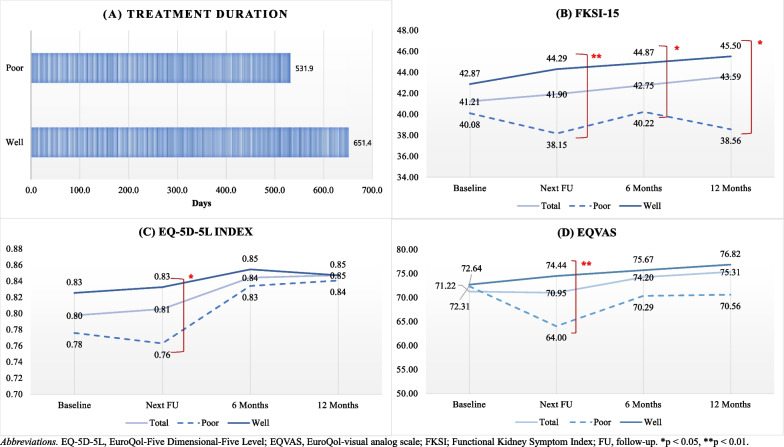
Univariate analysis comparing outcomes according to patients’ self-management of adverse events

Mean treatment duration for all patients was 621.3 (sd 501.8) days (Fig. 2). Treatment duration was longer in the `well managed’ group than in the `poor managed’ group (651.4 days vs 531.9 days, *p* = 0.292). The trend was maintained in multivariate analysis, with a coefficient value of 18 in the `well managed’ group compared to the `poor managed’ group, although there was no statistically significant difference (Table 4).

**Table 4:** Multivariate analysis for comparison of outcomes according to patients’ self-management of AEs

Outcomes	Patients’ self-management of AEs	Next follow-up (N = 71)			6 months (N = 47)			12 months (N = 30)		
		Coeff.	SE	*p* value	Coeff.	SE	*p* value	Coeff.	SE	*p* value
FKSI-15	Well (ref. poor)	4.16	1.46	0.006^a^	0.31	2.24	0.891^b^	1.88	3.69	0.619^c^
EQ-5D-5L	Well (ref. poor)	0.03	0.02	0.108^d^	-0.02	0.03	0.444^e^	< −0.01	0.03	0.883^f^
EQVAS	Well (ref. poor)	8.79	3.26	0.009^ g^	-9.19	5.19	0.087^ h^	7.32	4.60	0.138^i^
Treatment duration	Well (ref. poor)	NA	NA	NA	NA	NA	NA	18.06	168.80	0.915^j^

The *p* values of ‘a to i’ were estimated using sequential conditional mean models, adjusted with the following variables other than the fixed confounding variables, presenting R squares which mean each explanatory power of the models: ^a^FKSI-15 and ECOG PS at baseline, R^2^ = 78%; ^b^FKSI-15 at next follow-up, treatment duration, disease duration, ECOG PS at baseline and 6 months, and metastasis site at 6 months (bone), R^2^ = 65%; ^c^FKSI-15 at 6 months, disease duration, ECOG PS at 6 months and 12 months, and intervention schedule change at 12 months, R^2^ = 76%; ^d^EQ-5D-5L at baseline, BMI, histology, ECOG PS at baseline, and metastasis site at baseline (bone, brain, and pancreas), R^2^ = 72%; ^e^EQ-5D-5L at next follow-up and metastasis site at 6 months (lung, bone), R^2^ = 60%; ^f^EQ-5D-5L at 6 months, metastasis site at 6 months (LN), and ECOG PS at 12 months, R^2^ = 85%; ^g^EQVAS at baseline, BMI, and metastasis site at baseline (bone, LN, brain), R^2^ = 65%; ^h^EQVAS at next follow-up, BMI, disease duration, operation, and metastasis site at baseline (lung) and at 6 months (bone), R^2^ = 65%; and ^i^EQVAS at 6 months, disease duration, metastasis site at 12 months (LN, bone), and response criteria at 12 months, R^2^ = 72%

The *p* value of j was estimated using a linear regression model adjusted with the following variables other than the fixed confounding variables: response criteria at 6 months and metastasis site at 6 months (brain)

BMI, body mass index; coeff., coefficient; EQ-5D-5L, EuroQol-Five Dimensional-Five Level; EQVAS, EuroQol-visual analog scale; ECOG PS, Eastern Cooperative Oncology Group performance status; FKSI-15, Functional Kidney Symptom Index-15; LN, lymph node; NA, not applicable

Mean FKSI-15, EQ-5D-5L, and EQVAS scores at baseline for all patients were 41.2, 0.8, and 71.2, respectively, which increased slightly at 12 months to 43.6, 0.9, and 75.3, respectively. The differences over time were, however, not statistically significant in a linear mixed model (Fig. 2).

Differences in mean FKSI-15, EQ-5D-5L, and EQVAS scores at 6 months and 12 months according to patients’ management of AEs from univariate analysis, are presented in Fig. 2. Scores for the three PROs at the next follow-up visit (median 28 days, range 12 to 84 days) were significantly higher in the `well managed’ group than in the `poor managed’ group (p < 0.05). The trend towards a significant difference in FKSI-15 scores was retained throughout the 12-month study, although the statistical significance in EQ-5D-5L and EQVAS scores disappeared from 6 months. In multivariable SCMMs, patients’ self-management of AEs was positively associated with FKSI-15 and EQVAS at the next follow-up visit, in which the `well managed’ group reported higher scores, by 3.8 (*p* = 0.006) and 7.6 (*p* = 0.009) in the FKSI-15 and EQVAS, respectively, compared with the `poor managed’ group (Table 4).

## Discussion

This is the first real-world study to investigate patients’ self-management of seven frequently observed AEs related to targeted agents, and to explore PROs according to the status of self-management of AEs, in patients with advanced RCC in Korea.

The 77 enrolled patients experienced a mean of 2.8 cases of AEs per person and 231 cases of AEs in total. These were lower than those reported in a previous clinical trial but higher than those reported in a real-world study, which may be explained by the different observational periods among the studies [15, 16].

A total of 210 cases of the seven AEs of interest were observed. Most of these AEs were manageable (grades 1 to 2) and only a few cases were severe, similar to previous reports [17]. Within a short period to the next follow-up appointment (median 28 days), about 20% of cases were resolved and 14% of cases were improved. Meanwhile, 7% cases worsened in symptom grade. In a previous real-world chart review study, more than half (56%) of the AEs related to first-line TKIs were resolved or resolving at 3.5 months after onset [16]. More cases of worsened symptoms were, in particular, observed for fatigue in the current study. Symptoms should always be investigated for underlying causes, such as hyperthyroidism or anemia. Furthermore, fatigue may be exacerbated by underlying dehydration. Strengthening of patient education on nutrition, or consultation with a nutritionist, could help patients to alleviate their symptoms [18].

In our study, treatment schedule changes, including dose reduction, were observed less frequently in the AE `well managed’ group than in the `poor managed’ group, which may have resulted in the longer treatment duration observed in the AE `well managed’ group. A previous study showed a correlation between AEs and lower dose intensity and shorter survival times in patients with advanced RCC [19], suggesting that appropriate management of AEs is important for obtaining better treatment outcomes.

The key drivers of HRQoL deterioration in patients with mRCC are disease symptoms [20]. First-line systemic therapy does not change the HRQoL of patients with mRCC before progression [20]. In fact, complete/partial response or stable disease as the best response to targeted agents, rather than disease progression, have been shown to be associated with less deterioration of HRQoL in mRCC patients [21]. In our study, mean FKSI, EQ-5D, and EQVAS values did not change significantly because we excluded patients who had disease progression in the analysis for PROs. Interestingly, the mean values of those PROs were changed by the patients’ self-management of AEs. In this study, 71.9% of AE cases amongst a total of 210 AE cases were well managed by patients and 63% of all patients were in the well managed group (i.e. no poor/very poor AEs). The `well managed’ group showed higher FKSI and EQVAS scores, by 4.2 and 8.8 points, respectively, than the `poor managed’ group. These results strengthen the previous report of positive experiences of patients with mRCC with the recommendations for self-management of AEs while receiving targeted therapy [4]. However, although education for self-management of AEs such as management of hygiene and eating habits is likely to be provided at the start of treatment, strategies other than pharmacologic AE management are provided insufficiently in real-world practice; thus, repeated education reinforcing patients’ self-management of AEs could improve outcomes in advanced RCC patients treated with targeted therapy [16].

This study has several limitations. Firstly, the number of patients in this study may not be sufficient to reliably detect differences in outcomes. In addition, the final remission status of AEs was not investigated, which may alter the PROs at 6 months and 12 months. The number of patients observed decreased over time as this study was conducted under the schedule of usual clinical practice without any interventions. Therefore, circumspection is required in the interpretation and generalization of the results. Furthermore, we did not perform validation of the self-administered AE questionnaire used in this study as this was not a study objective. To use the same instrument in another study to compare the results between studies, validation of the questionnaire is required. Lastly, both symptom duration and perceptions of self-management, which may be associated with symptom severity and number, could confound the relationship between self-management of adverse events and outcomes; however, they were not considered in this study. As we developed the questionnaire on self-management of AEs based on patient education performed in real clinical practice, we think that the independent variable and its influence on outcomes in this study reflect the real situation of clinical practice.

Nevertheless, this study has remarkable strengths in that it is the first study to investigate treatment duration and PROs according to the status of patients’ AE management when treated with targeted therapy for advanced RCC; thus, it provides an in-depth understanding of patients’ AE management in real clinical practice. Moreover, as widely used validated measurements for PROs (both generic and disease specific) were used in this study, our results are a source for comparison with future studies in patients with the same or similar diseases.

## Conclusions

This prospective, longitudinal, observational study of patients with advanced RCC in Korea provides a special emphasis on the importance of patients’ self-management of AEs related to first targeted therapy. Patients in the well managed group showed significantly better improvement of PROs at the next-follow-up from the AE occurrence. Repeated education at every hospital follow-up visit for consolidating patients’ self-management of AEs could be considered in real-world clinical practice to enhance overall treatment outcomes while treating advanced RCC patients with first targeted therapy.

## Data Availability

The datasets used and analyzed during the current study are available from the corresponding author on reasonable request.

## References

[1] Motzer RJ, Jonasch E, Michaelson MD, Nandagopal L, Gore JL, George S, Alva A, Haas N, Harrison MR, Plimack ER, Sosman J, Agarwal N, Bhayani S, Choueiri TK, Costello BA, Derweesh IH, Gallagher TH, Hancock SL, Kyriakopoulos C, LaGrange C, Lam ET, Lau C, Lewis B, Manley B, McCreery B, McDonald A, Mortazavi A, Pierorazio PM, Ponsky L, Redman BG, Somer B, Wile G, Dwyer MA, Hammond LJ, Zuccarino-Catania G (2019). NCCN Guidelines Insights: Kidney Cancer, Version 2.2020. J Natl Compr Canc Netw.

[2] Miyake H, Harada K, Inoue TA, Fujisawa M (2014). Assessment of health-related quality of life in Japanese patients with metastatic renal cell carcinoma during treatment with tyrosine kinase inhibitors. Med Oncol.

[3] Méndez-Vidal MJ, Martínez Ortega E, Montesa Pino A, Pérez Valderrama B, Viciana R (2012). Management of adverse events of targeted therapies in normal and special patients with metastatic renal cell carcinoma. Cancer Metastasis Rev.

[4] Eberhardt-Wetherington B, Gall V, Lang M, Schmidinger M, Gruenwald V, Claussen C, Wartenberg M, Niedtner R, Kalanovic D (2013). Patient and physician perspective on published therapy management recommendations for TKI-treated patients with a focus on sunitinib: The TheMaPaC project (Therapy Management Patient Consensus). J Clin Oncol.

[5] Feinberg BA, Jolly P, Wang ST, Fortner B, Scott J, Gilmore J, Neary MP, Duh MS (2012). Safety and treatment patterns of angiogenesis inhibitors in patients with metastatic renal cell carcinoma: evidence from US community oncology clinics. Med Oncol.

[6] Chow SC, Liu JP (2004). Design and Analysis of Clinical Trials: Concepts and Methodologies.

[7] Common Terminology Criteria for Adverse Events (CTCAE) v4.0. https://ctep.cancer.gov/protocoldevelopment/electronic_applications/ctc.htm#ctc_40 15818867

[8] Herdman M, Gudex C, Lloyd A, Janssen M, Kind P, Parkin D, Bonsel G, Badia X (2011). Development and preliminary testing of the new five-level version of EQ-5D (EQ-5D-5L). Qual Life Res.

[9] Kim SH, Ahn J, Ock M, Shin S, Park J, Luo N, Jo MW (2016). The EQ-5D-5L valuation study in Korea. Qual Life Res.

[10] Bonomi AE, Cella DF, Hahn EA, Bjordal K, Sperner-Unterweger B, Gangeri L, Bergman B, Willems-Groot J, Hanquet P, Zittoun R (1996). Multilingual translation of the Functional Assessment of Cancer Therapy (FACT) quality of life measurement system. Qual Life Res.

[11] Eremenco SL, Cella D, Arnold BJ (2005). A comprehensive method for the translation and cross-cultural validation of health status questionnaires. Eval Health Prof.

[12] Wild D, Grove A, Martin M, Eremenco S, McElroy S, Verjee-Lorenz A, Erikson P, ISPOR Task Force for Translation and Cultural Adaptation (2005). Principles of good practice for the translation and cultural adaptation process for patient reported outcomes (PRO) measures: report of the ISPOR Task Force for Translation and Cultural Adaptation. Value Health.

[13] Cella D, Yount S, Du H, Dhanda R, Gondek K, Langefeld K, George J, Bro WP, Kelly C, Bukowski R (2006). Development and validation of the functional assessment of cancer therapy-kidney symptom index (FKSI). J Support Oncol.

[14] Keogh RH, Daniel RM, VanderWeele TJ, Vansteelandt S (2018). Analysis of longitudinal studies with repeated outcome measures: adjusting for time-dependent confounding using conventional methods. Am J Epidemiol.

[15] Motzer RJ, Hutson TE, Cella D, Reeves J, Hawkins R, Guo J, Nathan P, Staehler M, de Souza P, Merchan JR, Boleti E, Fife K, Jin J, Jones R, Uemura H, De Giorgi U, Harmenberg U, Wang J, Sternberg CN, Deen K, McCann L, Hackshaw MD, Crescenzo R, Pandite LN, Choueiri TK (2013). Pazopanib versus sunitinib in metastatic renal-cell carcinoma. N Engl J Med.

[16] Srinivas S, Stein D, Teltsch DY, Tao S, Cisar L, Ramaswamy K (2018). Real-world chart review study of adverse events management in patients taking tyrosine kinase inhibitors to treat metastatic renal cell carcinoma. J Oncol Pharm Pract.

[17] Eisen T, Sternberg CN, Robert C, Mulders P, Pyle L, Zbinden S, Izzedine H, Escudier B (2012). Targeted therapies for renal cell carcinoma: review of adverse event management strategies. J Natl Cancer Inst.

[18] Kollmannsberger C, Soulieres D, Wong R, Scalera A, Gaspo R, Bjarnason G (2007). Sunitinib therapy for metastatic renal cell carcinoma: recommendations for management of side effects. Can Urol Assoc J.

[19] Porta C, Levy A, Hawkins R, Castellano D, Bellmunt J, Nathan P, McDermott R, Wagstaff J, Donnellan P, McCaffrey J, Vekeman F, Neary MP, Diaz J, Mehmud F, Duh MS (2014). Impact of adverse events, treatment modifications, and dose intensity on survival among patients with advanced renal cell carcinoma treated with first-line sunitinib: a medical chart review across ten centers in five European countries. Cancer Med.

[20] de Groot S, Redekop WK, Versteegh MM, Sleijfer S, Oosterwijk E, Kiemeney LALM, Uyl-de Groot CA (2018). Health-related quality of life and its determinants in patients with metastatic renal cell carcinoma. Qual Life Res.

[21] Cella D, Pickard AS, Duh MS, Guerin A, Mishagina N, Antras L, Neary MP, McCann L, Hodge R, Sternberg CN (2012). Health-related quality of life in patients with advanced renal cell carcinoma receiving pazopanib or placebo in a randomised phase III trial. Eur J Cancer.

